# Photobiomodulation Modulates the Response of Zoledronic-Acid-Treated Osteoblast-like SaOs-2 Cells: Implications for Bisphosphonate-Related Osteonecrosis

**DOI:** 10.3390/bioengineering13010088

**Published:** 2026-01-12

**Authors:** Rodrigo Antonio Carvalho Andraus, Ana Flávia Spadaccini Silva de Oliveira, Mário Celso Teixeira Lopes, Diego César Marques, Vanessa Gabriela Gonzales Marques, Deise Aparecida de Almeida Pires de Oliveira, Rodrigo Franco de Oliveira, Orlando Aguirres Guedes, Helder Fernandes de Oliveira, João Pedro Ribeiro Afonso, Iransé Oliveira Silva, Luiz Vicente Franco de Oliveira, Claudia Santos Oliveira, Regina Célia Poli, Luciana Prado Maia

**Affiliations:** 1 Graduate Program in Human Movement and Rehabilitation, Evangelical University of Goias (UniEVANGÉLICA), Anápolis 75083-515, GO, Brazil; rodrigoandraus@gmail.com (R.A.C.A.); drdiegobucomaxilo@gmail.com (D.C.M.); dravanessagonzalessmsipora@gmail.com (V.G.G.M.); deise.oliveira@unievangelica.edu.br (D.A.d.A.P.d.O.); rodrigo.oliveira@unievangelica.edu.br (R.F.d.O.); joaopedro180599@gmail.com (J.P.R.A.); iranse.silva@unievangelica.edu.br (I.O.S.); oliveira.lvf@uninove.br (L.V.F.d.O.); claudia.oliveira@unievangelica.edu.br (C.S.O.); 2 School of Physiotherapy, University Center UNIFIO, Ourinhos 19915-501, SP, Brazil; ana_spadaccini@hotmail.com; 3 Graduate Program in Dentistry, Evangelical University of Goias (UniEVANGÉLICA), Anápolis 75083-515, GO, Brazil; mariocelsotl@gmail.com (M.C.T.L.); orlando.guedes@unievangelica.edu.br (O.A.G.); helder.oliveira@unievangelica.edu.br (H.F.d.O.); 4 Graduate Program in Rehabilitation Sciences, University Pitagoras UNOPAR, Londrina 86041-140, PR, Brazil; regina.frederico@unopar.br

**Keywords:** laser therapy, osteosarcoma, photobiomodulation, apoptosis, gene expression

## Abstract

This study aimed to evaluate the effects of laser photobiomodulation (PBM) therapy in SaOs-2 osteosarcoma cells treated with zoledronic acid (ZA), a bisphosphonate, in vitro, mimicking a bisphosphonate-related osteonecrosis of the jaw (BRONJ) situation. Cells were treated with 100 μM ZA for 24 h and subjected to PBM using wavelengths of 660 nm and 808 nm at energy delivered of 1, 5, 10, and 20 J. After 24 h, metabolic activity, apoptosis, and BAX and BCL-2 gene expression were analyzed. Data were compared using one-way ANOVA followed by Tukey’s post hoc test (*p* < 0.05). ZA significantly reduced metabolic activity (*p* < 0.05), an effect attenuated by PBM at 808 nm with 1 J, while BCL-2 expression increased with 1 J at 660 nm and with 1 J and 20 J at 808 nm. However, PBM did not reverse ZA-induced apoptosis. In conclusion, PBM modulated the response of SaOs-2 osteoblastic cells treated with ZA in a wavelength- and dose-dependent manner. PBM at 808 nm and 1 J stimulated cell metabolic activity and upregulated BCL-2 expression, suggesting a potential protective effect against ZA-induced cytotoxicity.

## 1. Introduction

Cancer cells located in the bone marrow induce bone disease through interactions with local bone cells, producing factors that promote the development and activity of osteoclasts. Bone tissue serves as an abundant reservoir of inactive growth factors that are activated and/or released during bone resorption, thereby stimulating tumor cell proliferation [1,2]. Consequently, cancer cells and osteoclasts become engaged in a self-perpetuating cycle.

Patients with skeletal system disorders such as osteoporosis [3,4,5] and Paget’s disease [6,7], as well as those with bone tumors, bone metastases [8] and specific cancers including breast [9,10] and prostate cancer [11,12], are frequently treated with bisphosphonates—a class of antiresorptive drugs designed to target bone-destructive activity and disrupt this cycle [1]. However, although bisphosphonates are effective in suppressing osteoclastic bone resorption, prolonged administration has been linked to impaired bone healing and the occurrence of osteonecrosis of the jaw (ONJ) [13].

Bisphosphonate-related osteonecrosis of the jaw (BRONJ) is a debilitating disorder characterized by areas of exposed necrotic bone in the maxillofacial region, which markedly reduces patients’ quality of life. The pathogenesis of BRONJ has been widely investigated and is believed to be associated with the inhibition of bone remodeling resulting from osteoclast suppression, potentially leading to bone sclerosis and ischemia [14,15,16]. Impaired angiogenesis and microbial infections have been suggested as contributing factors. BRONJ remains a difficult condition to manage due to its refractory behavior, and despite various proposed treatment protocols, an optimal therapeutic strategy has yet to be established [17,18].

There is increasing scientific interest in the potential role of laser photobiomodulation (PBM) therapy as an adjunctive approach for the prevention and management of BRONJ [19,20], owing to its effects on wound healing, inflammation modulation, angiogenesis, and bone regeneration [21,22,23]. As bisphosphonates inhibit bone remodeling and angiogenesis—two fundamental processes in bone and mucosal healing— PBM may help mitigate these adverse effects by enhancing cellular activity and promoting tissue repair.

The mechanism of laser PBM action involves the stimulation of the mitochondrial respiratory chain, leading to increased production of adenosine triphosphate (ATP) and the release of specific levels of reactive oxygen species (ROS) and nitric oxide (NO). The release of NO induces vasodilation, enhances microcirculation, and facilitates tissue repair processes [23,24,25]. Similarly, ROS release can trigger transcriptional changes and activate nuclear factor κB (NF-κB), which promotes the synthesis of anti-apoptotic proteins while simultaneously stimulating cell proliferation and migration [26].

Bone regeneration is a dynamic process that depends on a delicate balance between osteoblast and osteoclast activity, in which apoptosis plays a key regulatory role. Under normal physiological conditions, tissue homeostasis is maintained by the equilibrium between anti-apoptotic and pro-apoptotic proteins. Disruption of this balance—for instance, due to bisphosphonate-induced inhibition of bone turnover—may lead to reduced cell viability and impaired bone remodeling [27]. Among the BCL family proteins that regulate apoptosis, BCL-2 and BAX are particularly important in bone metabolism. BCL-2 acts as an anti-apoptotic factor that preserves mitochondrial integrity, promoting osteoblast survival and contributing to bone formation, whereas BAX functions as a pro-apoptotic factor that, when overexpressed, induces mitochondrial damage and triggers programmed cell death [28,29,30]. Therefore, the relative expression of these proteins reflects the balance between cell survival and apoptosis, influencing bone regeneration capacity and potentially mediating the effects of PBM in bisphosphonate-treated cells.

Several studies have explored the use of PBM in the management of BRONJ, emphasizing its potential to alleviate pain, promote soft tissue healing, and stimulate bone regeneration [31,32]. Preliminary evidence indicates that PBM may serve as a valuable adjunctive therapy, particularly when combined with conventional approaches such as surgical debridement and pharmacological treatment. Nonetheless, despite the growing interest in this modality, the absence of standardized laser parameters limits the comparability of findings across studies [33,34,35]. Variations in study design, laser parameters, treatment protocols, and outcome assessments further complicate data interpretation. Additionally, the underlying biological mechanisms in the healing process associated with PBM in bone cells treated with bisphosphonates remain not fully understood.

Therefore, the aim of the present study was to evaluate the effects of PBM on cell metabolic activity, apoptosis, and expression of apoptotic genes in osteosarcoma Saos-2 cells treated with the bisphosphonate zoledronic acid (ZA).

It is important to emphasize that this study was designed as an exploratory in vitro investigation and reflects conditions relevant to BRONJ, where maxillary and mandibular bone surfaces are chronically exposed to bisphosphonates and may benefit from photobiomodulation-induced modulation of osteoblast-like cellular responses. The clinical context motivating this work is the impaired bone healing observed in BRONJ lesions of the maxilla and mandible, which are accessible to PBM irradiation.

## 2. Materials and Methods

### 2.1. Cell Culture

SaOs-2 human osteoblastic cells (Rio de Janeiro Cell Bank—BCRJ, Rio de Janeiro, Brazil) were cultured in McCoy’s 5A culture medium (GIBCO^TM^—Invitrogen Corporation, Grand Island, New York, NY, USA) supplemented with 10% fetal bovine serum (FBS—GIBCO) and 1% antibiotic-antimycotic solution (GIBCO). The cells were maintained in a humidified incubator with 5% CO_2_ at 37 °C. Upon reaching approximately 80% subconfluence, the cells were subcultured and seeded into 96-well plates (Techno Plastic Products AG TPP—Zollstrasse, Trasadingen, Switzerland) at a density of 1 × 10^4^ cells per well and allowed to adhere for 24 h before treatment.

### 2.2. Determination of Ideal Concentration of ZA

After 24 h of adherence, the cells were incubated with ZA (Zometa^®^—Novartis, Monções, Brazil) at concentrations of 1, 5, 10, 25, 50, and 100 μM, to determine the concentration capable of reducing metabolic activity by up to 30%, since a greater reduction indicates cytotoxicity [36]. In this study, ‘metabolic activity’ refers specifically to the reduction in MTT (3-(4,5-dimethyltriazol-2yl)-2,5-diphenyl tetrazoline bromide) by mitochondrial and cytosolic dehydrogenases, which reflects cellular metabolic status but does not directly assess clonogenic survival or proliferation, as previously demonstrated in methodological evaluations [37,38]. Culture medium was used as a negative control, and 30% hydrogen peroxide served as a positive control. The MTT assay (SIGMA-Aldrich, Co, 3050 Spruce St, St. Louis, MO, USA) was used to assess ZA cytotoxicity at 24, 48, and 72 h, to allow controlled in vitro exposure. Exposure times were selected based on previous pharmacokinetic evidence that ZA exerts prolonged cellular effects despite its rapid plasma clearance, thereby supporting the relevance of these in vitro incubation periods [39].

### 2.3. Laser Photobiomodulation Therapy (LPT)

SaOs-2 treated with ZA at the concentration determined for 24 h were treated with a single application of PBM. The laser device used was the Laser Therapy EC (Thera Laser DMC Equipment Ltda, São Carlos, Brazil), which was fixed on a support, while the plate was positioned to direct irradiation toward the wells. Cells were plated with spaces between wells, and Teflon separators were placed in empty wells to prevent energy absorption in adjacent wells by reflection [40]. This material was chosen due to its combination of chemical, thermal, and mechanical stability, high fractional free volume, low refractive index, low surface energy, and broad optical transparency [41]. The plate lid was also covered with a black film to ensure that the irradiation was directed specifically to the treatment well.

The parameters used are summarized in Table 1, which presents energy delivered of 1, 5, 10, and 20 J at wavelengths of 660 and 808 nm. Cells cultured in standard medium served as a negative control, whereas cells treated with ZA without irradiation served as the positive control. These cells were maintained under the same conditions as irradiated cells. The experimental groups were defined as described in Table 2.

### 2.4. Metabolic Activity Assessment—MTT Assay

The MTT assay (SIGMA-Aldrich) was used to assess metabolic activity 24 h after PBM. Aliquots of MTT solution (5 mg/mL in phosphate-buffered saline [PBS]; Gibco) were prepared. The primary cultures were incubated with this solution, diluted to 10% in culture medium, for 4 h at 37 °C in a humidified atmosphere containing 5% CO_2_. After incubation, the supernatant was removed, and 100 μL of dimethylsulfoxide (DMSO; SIGMA-Aldrich) was added to each well. The plates were gently agitated for 5 min to solubilize the formazan crystals. Absorbance was then measured at a wavelength of 570 nm using a microplate spectrophotometer (Thermo Fisher Scientific—Multiskan FC Microplate Photometer—51119000).

Metabolic activity was expressed as a percentage relative to the negative control, according to the following formula:

$$Metabolic\;activity\;\left(\%\right)=\left(\frac{ODt-ODb}{ODc-ODb}\right)*100$$

where *OD* = optical density; *t* = treatment; *b* = blank; *c* = control.

This experiment was performed in triplicate.

### 2.5. Analysis of Cell Apoptosis—Flow Cytometry

Cell apoptosis was evaluated 24 h after PBM. Briefly, treated cells were stained with 10 μL of Annexin V-FITC and 2 μL propidium iodide in 200 μL cell suspension, according to the manufacturer’s instructions (FITC Annexin V Dead Cell Apoptosis Kit with FITC Annexin V and Propidium Iodide; Invitrogen Corp., Carlsbad, CA, USA). Fluorescence acquisition of 5000 events was performed on a BD Accuri C6^®^ flow cytometer (BD Biosciences, San Jose, CA, USA). Data were analyzed with BD Accuri C6^®^ software (v1.1), and cells were classified as follows: viable cells (unstained; AV^−^/PI^−^; Q4-LL), early apoptotic cells (Annexin V–positive only; AV^+^/PI^−^, Q4–LR), late apoptotic cells (Annexin V– and PI–positive; AV^+^/PI^+^, Q4–UR), and necrotic cells (PI–positive only; AV^−^/PI^+^, Q4–UL).

This experiment was conducted using a single pooled sample. This methodological approach was considered appropriate because flow cytometry is a highly sensitive and accurate technique, allowing for reliable assessment of cell populations even with pooled samples. Obtaining an adequate cell number from individual wells was not feasible; therefore, the samples were combined to provide sufficient material for flow cytometry analysis. This procedure ensured representative analysis while maintaining experimental consistency.

### 2.6. Gene Expression

Twenty-four hours after irradiation, the culture medium was removed from the wells, and total RNA extraction was performed using the Cells-to-cDNA^TM^ II Kit (Thermo Fisher Scientific Inc., Waltham, MA, USA), according to the manufacturer’s instructions. The total RNA concentration and purity were quantified using a NanoDrop Lite^®^ spectrophotometer (Thermo Fisher Scientific Inc.).

Real-time polymerase chain reaction (RT-qPCR) was carried out using Fast Sybr^®^ green Master Mix reagent (Applied Biosystems, Waltham, MA, USA) on a StepOnePlus^TM^ Real-Time PCR System (Applied Biosystems, USA). Gene expression levels were normalized to GAPDH as the housekeeping gene. The primer sequences used in this study are listed in Table 3.

Reactions were performed in a final volume of 10 µL containing 5 µL of Fast Sybr^®^ green Master Mix (Applied Biosystems, USA), 0.2 µL of each primer (Applied Biosystems, USA) (Table 3), 2.6 µL of ultrapure H_2_O, and 2 µL of cDNA. Amplification was carried out under the following cycling conditions: initial denaturation at 95 °C for 20 s, followed by 40 cycles of 95 °C for 3 s and 60 °C for 30 s. Relative gene expression levels were calculated using the 2^−^ΔΔCt method, and results are expressed as fold change relative to the control group.

This experiment was conducted in duplicate. This methodological approach was deemed appropriate because RT-qPCR is a highly sensitive and reproducible technique, allowing for reliable quantification even with duplicate measurements. RNA extraction from individual wells was not feasible due to the limited amount of cellular material available in each well; therefore, the samples were pooled to obtain sufficient RNA yield for RT-qPCR analysis. Pooling was performed within each experimental group to maintain sample integrity and comparability.

### 2.7. Statistical Analysis

Data normality was assessed using the Shapiro–Wilk test. One-way ANOVA followed by the Dunnett’s test was applied to evaluate differences among groups in cell metabolic activity, with the significance level set at 5% (*p* < 0.05). Statistical analyses were performed using GraphPad Prism version 6 (GraphPad Software, San Diego, CA, USA). For apoptosis and gene expression assays, descriptive statistics were presented due to methodological constraints.

## 3. Results

### 3.1. Determination of Ideal Concentration of ZA

**Figure 1:**
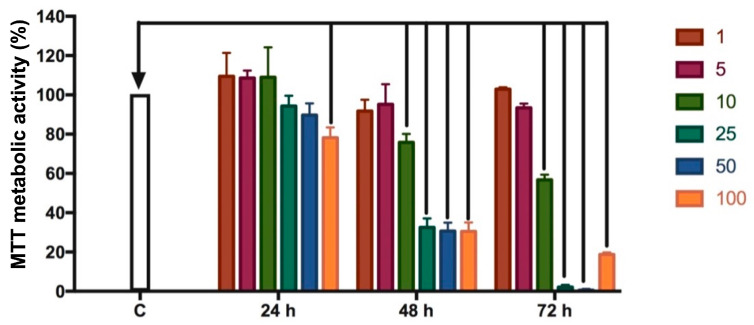
Mean and standard deviation of SaOs-2 metabolic activity (%) following treatment with 1, 5, 10, 25, 50, and 100 μM of zoledronic acid (ZA) for 24, 48, and 72 h. Arrows indicate statistically significant differences (*p* < 0.0001) compared to the control group (C).

Regarding the ZA concentration (Figure 1), after 24 h, only the 100 µM dose resulted in a significant cytotoxic effect compared to the control group (*p* < 0.0001), although the reduction in metabolic activity was limited to approximately 20%. After 48 h, a marked cytotoxicity was observed in the groups treated with 10, 25, 50, and 100 µM, with the three highest concentrations (25, 50, and 100 µM) reducing metabolic activity by more than 50% (*p* < 0.0001). At the lower concentrations (1 and 5 µM), no significant reductions were detected. After 72 h, an even greater decrease was observed at 10, 25, 50, and 100 µM, whereas the lowest doses (1 and 5 µM) continued to show no effect. Therefore, the 10 µM concentration was selected as the ideal dose, as it reduced metabolic activity by approximately 30% after 48 h of treatment.

### 3.2. Metabolic Activity Assessment

**Figure 2:**
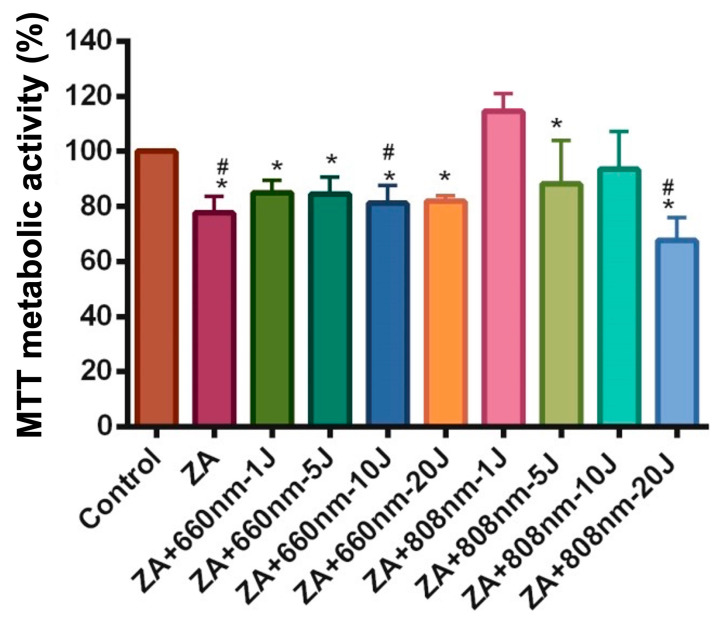
Mean and standard deviation of SaOs-2 metabolic activity (%) after treatment with 10 µM zoledronic acid (ZA) for 24 h and a single application of photobiomodulation (PBM) using 660 nm and 808 nm wavelengths at energy delivered of 1, 5, 10, and 20 J. The tic-tac-toe symbol (#) indicates a statistically significant difference compared with the control group (*p* < 0.05), whereas the asterisk (*) indicates a statistically significant difference compared with the ZA+808nm-1J group (*p* < 0.0001).

Metabolic activity results are shown in Figure 2. Treatment with ZA led to an average reduction of 22.36% in metabolic activity compared with the control group (*p* = 0.0025). The ZA+660nm-10J and ZA+808nm-20J groups also showed significant reductions—18.81% and 32.43%, respectively—relative to the control group (*p* < 0.05), with no significant differences when compared with the ZA group (*p* > 0.05). Additionally, the ZA+660nm-1J, ZA+660nm-5J, ZA+660nm-20J, ZA+808nm-5J, and ZA+808nm-10J groups showed no significant differences compared with either the control or ZA groups (*p* > 0.05). Interestingly, the ZA+808 nm-1J group exhibited significantly higher metabolic activity compared with the ZA group (*p* < 0.0001), as well as relative to all red laser–irradiated groups and the ZA+808nm-5J and ZA+808nm-20J groups.

### 3.3. Cell Apoptosis

**Figure 3:**
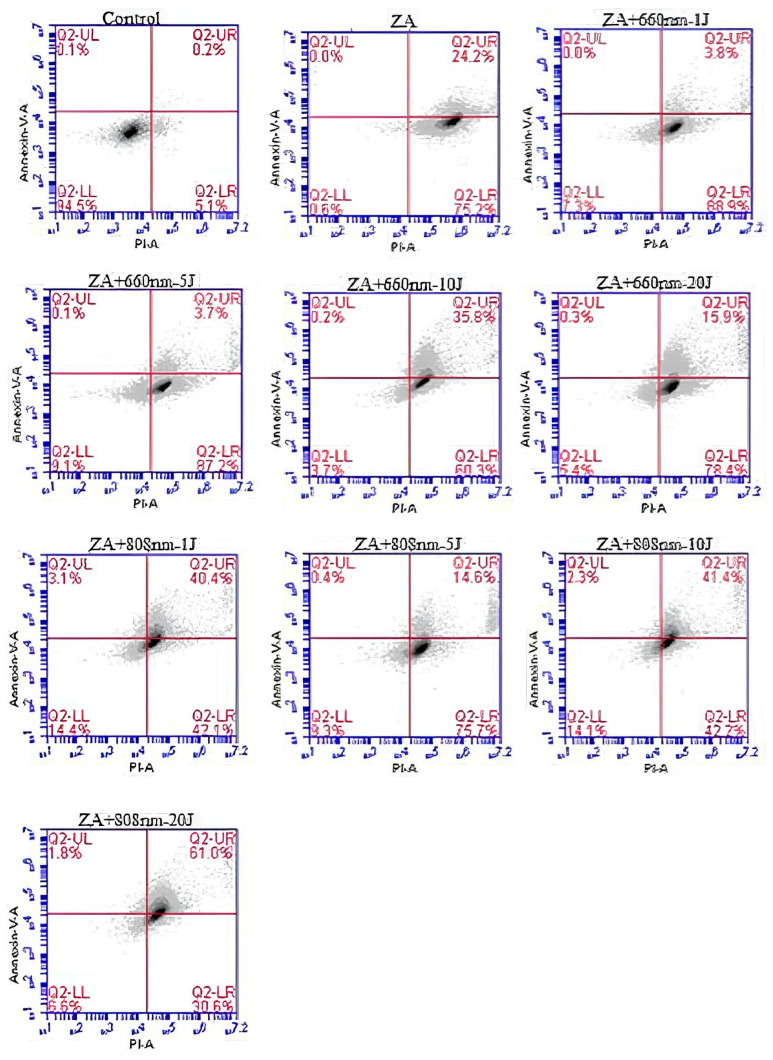
Distribution of SaOs-2 cells according to cell death stage. Viable cells (AV^−^/PI^−^; Q2-LL), early apoptosis (AV^+^/PI^−^; Q2-LR), late apoptosis (AV^+^/PI^+^; Q2-UR), and necrosis (AV^−^/PI^+^; Q2-UL) in osteosarcoma cells treated with 10 µM zoledronic acid (ZA) for 24 h and with a single application of photobiomodulation (PBM) and at wavelengths of 660 nm and 808 nm and energy delivered of 1, 5, 10, and 20 J.

**Figure 4:**
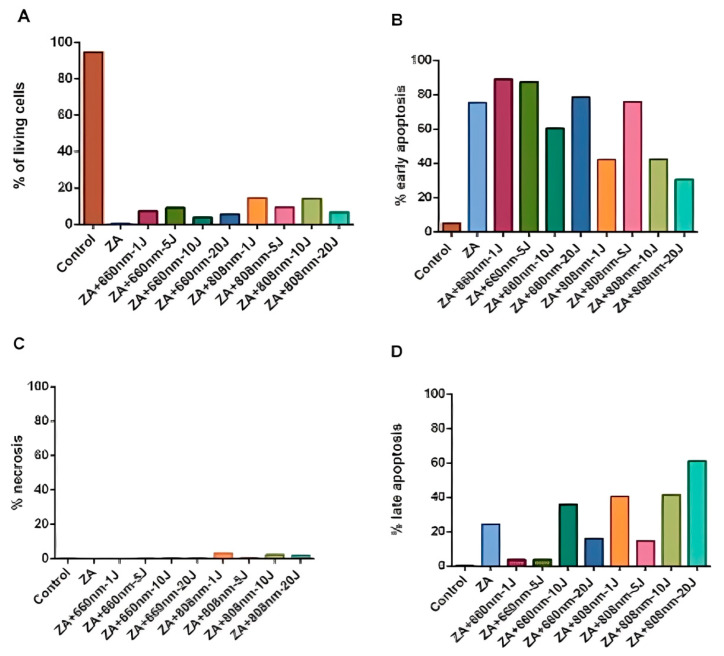
(**A**) Percentage of living cells (unlabeled), (**B**) cells in early apoptosis (Annexin V–labeled), (**C**) necrotic cells (PI-labeled), and (**D**) cells in late apoptosis (double-labeled with Annexin V and PI) in osteosarcoma cells treated or not (control) with photobiomodulation (PBM) and zoledronic acid (ZA) at wavelengths of 660 nm and 808 nm and energy doses of 1, 5, 10, and 20 J.

In the apoptosis assay (Figure 3 and Figure 4), treatment with ZA reduced the proportion of viable cells compared with the control group, and photobiomodulation (PBM) did not attenuate this effect in any of the experimental groups evaluated (Figure 4A). Higher proportions of cells in early apoptosis were observed in the ZA, ZA+660nm-1J, ZA+660nm-5J, ZA+660nm-20J, and ZA+808nm-5J groups (75.2%, 88.9%, 87.2%, and 78.4%, respectively) (Figure 4B).

### 3.4. Gene Expression

**Figure 5:**
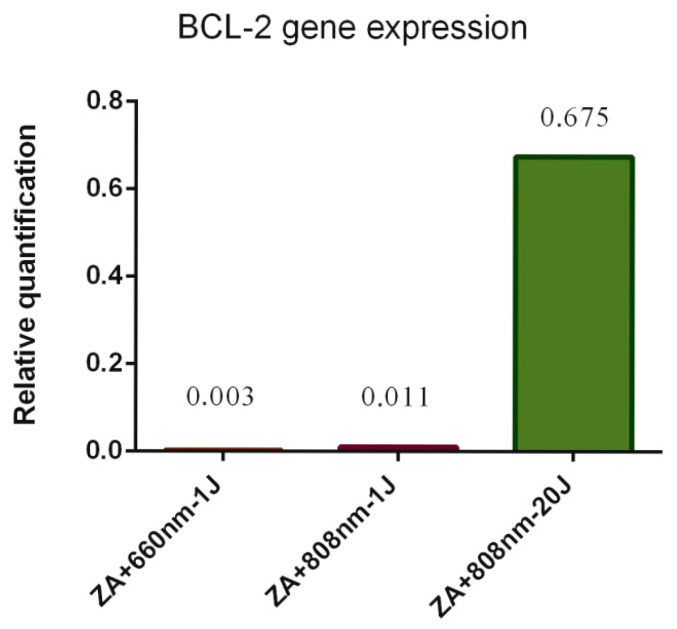
Gene expression of SaOs-2 cells treated with zoledronic acid (ZA) and photobiomodulation (PBM) in the ZA+660nm-1J, ZA+808nm-1J, and ZA+808nm-20J groups.

Regarding gene expression, BCL-2 expression was detected in ZA+660nm-1J, ZA+808 nm-1J, and ZA+808nm-20J groups, and all of which increased relative expression of this gene (Figure 5). In contrast, BAX gene expression was not detected in any of the experimental groups. As no gene expression was detected in the remaining experimental groups, these results were omitted from the analysis for clarity.

## 4. Discussion

The present study investigated the effects of photobiomodulation (PBM) on SaOs-2 osteoblastic cells treated with zoledronic acid (ZA), focusing on metabolic activity, apoptosis, and gene expression. The results demonstrated that ZA at a concentration of 10 µM reduced metabolic activity by approximately 30% after 48 h of incubation. PBM influenced the biological response of ZA-treated cells depending on the irradiation parameters. Specifically, irradiation at 808 nm with an energy dose of 1 J stimulated metabolic activity and upregulated the anti-apoptotic gene BCL-2, while other irradiation protocols did not significantly modify metabolic activity or apoptosis patterns. Flow cytometry revealed that ZA alone and in combination with PBM increased the proportion of cells in early apoptosis, and no expression of the BAX gene was detected in any experimental group. Because this is an exploratory in vitro study, our results should not be interpreted as an attempt to irradiate osteosarcoma cells or bone marrow in vivo. Instead, the cellular model was chosen to mimic osteoblast-like behavior under bisphosphonate exposure, which is a relevant pathological condition in BRONJ lesions affecting the maxillofacial skeleton.

Regarding the effect of ZA on tumor cells, no evaluated concentration was cytotoxic after 24 h of treatment, considering that a cytotoxic effect requires a reduction of at least 30% in cell metabolic activity compared with the control group [36]. This effect was observed after 48 h at concentrations equal to or greater than 10 µM, which was therefore considered the ideal concentration to achieve up to a 30% reduction in metabolic activity. Similar results were reported by Castro et al. [42] and Ravosa et al. [43], who evaluated the effects of ZA on osteoblastic and epithelial cell lines, respectively, and demonstrated that ZA was cytotoxic, decreasing cell metabolic activity and inducing cell death—particularly at a concentration of 10 µM.

It is important to emphasize that, although SaOs-2 cells are derived from osteosarcoma, the present in vitro model does not aim to simulate direct irradiation of intramedullary tumors. Rather, it serves as an osteoblastic-like cellular model to investigate how PBM modulates the response of bone-related cells to zoledronic acid under controlled laboratory conditions. In addition, the 24 h exposure to zoledronic acid used in this study does not aim to simulate a 24 h continuous infusion in vivo. Instead, it reflects a standardized in vitro model widely adopted in studies evaluating the cellular effects of bisphosphonates [39]. Although plasma concentrations of ZA decline rapidly after infusion, the drug binds strongly to mineralized bone and remains biologically active for prolonged periods. Therefore, exposure times ranging from 24 to 72 h are commonly used to reproduce this sustained pharmacodynamic effect in vitro, as demonstrated in previous studies [39].

When analyzing the effects of PBM in ZA-incubated cells, the present study found that PBM at a wavelength of 808 nm and an energy dose of 1 J stimulated metabolic activity and increased expression of the BCL-2 gene, an important regulator of apoptosis. Supporting these findings, Akens et al. [44] reported that ZA at 10 μM reduced the metabolic activity of SaOs-2 cells by 50%, and that PBM at 690 nm with an energy density of 20 J/cm^2^ induced apoptosis in both ZA-incubated and control cells. Similarly, Pansani et al. [45] observed that PBM at 0.5 and 3 J/cm^2^ (780 nm) did not increase metabolic activity in ZA-treated cells, consistent with the present results. It is important to note that metabolic activity does not necessarily reflect cell viability, as treatments may alter metabolic function without affecting cell number or survival [1].

The apoptosis analysis also revealed an increase in early apoptosis in the group treated with ZA alone and in most groups treated with ZA combined with PBM. In contrast, Pansani et al. [45] reported a significant increase in apoptosis in epithelial cells and fibroblasts treated with ZA alone; however, no difference was observed when irradiated cells were compared to non-irradiated ZA-treated cells.

With respect to BCL-2 expression, no previous studies were found that directly evaluated this apoptotic marker in the context of PBM combined with ZA. Nevertheless, Karabulut et al. [46] demonstrated that ZA synergistically inhibited the growth of PC-3 and DU-145 cancer cells through downregulation of the anti-apoptotic protein BCL-2. Additionally, Wang et al. [47], in a study using three human cervical cancer cell lines (HeLa, SiHa, and CaSki), observed ZA-induced cell death characterized by apoptosis and autophagy, associated with changes in BCL-2 and BAX expression levels. These findings may help explain the increased BCL-2 expression observed in the present study.

The wavelengths used in this work (660 nm and 808 nm) fall within the well-defined optical therapeutic window commonly employed in photobiomodulation research. These wavelengths are absorbed primarily by mitochondrial chromophores, especially cytochrome-c oxidase, leading to modulation of cellular metabolism, ATP production, and redox signaling. Their biological effects in osteoblasts and osteoblast-like cells have been extensively documented in PBM literature [48].

This study presents certain limitations that should be acknowledged. First, the in vitro design, although controlled and reproducible, does not fully replicate the complex biological interactions that occur in vivo, such as systemic metabolism, vascularization, and immune modulation, which may influence the response to both zoledronic acid (ZA) and photobiomodulation (PBM). Additionally, although the 24–72 h ZA exposure period cannot perfectly mirror the dynamic pharmacokinetics of in vivo administration, it reflects the drug’s well-documented prolonged pharmacodynamic activity following systemic delivery, supporting its use in controlled in vitro assays. Importantly, the MTT assay used in this study measures mitochondrial metabolic activity, not true cell viability or proliferation, and no direct assays of proliferation or clonogenic survival were performed. The limited cellular yield per well required pooling of samples for flow cytometry and RT-qPCR analyses, which prevented statistical comparison between replicates, even though these are highly accurate assays. Furthermore, only two wavelengths (660 and 808 nm) and specific energy doses were tested, which may not encompass the full range of potentially therapeutic PBM parameters. Finally, the use of a single osteoblastic cell line (SaOs-2) restricts the generalizability of the findings to other bone cell types or in vivo conditions. Despite these limitations, the study provides relevant insights into the cellular response of osteoblastic cells to PBM in the presence of ZA and supports further investigations using additional models and irradiation protocols.

Schartinger et al. [49] demonstrated that identical PBM parameters can produce distinct cellular responses depending on the cell lineage exposed to irradiation. To date, little is known about the effects of PBM on bone cell cultures treated with bisphosphonates. The present findings indicate that PBM can modulate the metabolism of SaOs-2 osteoblastic cells. However, results from in vitro experiments are not easily comparable, as they depend on several factors, including irradiation parameters, cell-handling procedures, laboratory techniques, and irradiation timing [50].

Taken together, these results underscore the need to standardize PBM parameters for the treatment of bone alterations associated with bisphosphonate therapy. Therefore, when considering PBM as a potential therapeutic approach, further studies are essential to elucidate the main factors influencing cellular responses in this model and to optimize the use of this therapy in clinical applications.

## 5. Conclusions

Within the limitations of this in vitro study, photobiomodulation (PBM) demonstrated the ability to modulate the biological response of SaOs-2 osteoblastic cells incubated with zoledronic acid (ZA). ZA at a concentration of 10 µM for 24 h reduced cell metabolic activity by approximately 30%, confirming its cytotoxic potential at this dose. Among the irradiation parameters tested, PBM at 808 nm with an energy dose of 1 J stimulated cell metabolic activity and upregulated the anti-apoptotic BCL-2 gene, suggesting a potential protective cellular effect. However, other irradiation conditions did not significantly influence metabolic activity or apoptosis, and BAX expression was not detected in any experimental group. These findings indicate that the cellular response to PBM is wavelength- and dose-dependent, reinforcing the importance of parameter optimization when using PBM as an adjunct to manage bisphosphonate-related bone alterations. Further studies, particularly in vivo, are warranted to clarify the mechanisms involved and to define clinically relevant PBM protocols.

## Figures and Tables

**Table 1:** Irradiation parameters.

Beam spot size at target (cm^2^)	0.0324
Irradiance (mW/cm^2^)	3086.4
Time exposure (s)	10–50–100–200
Fluence (J/cm^2^)	33–166–333–666
Energy delivered (J)	1–5–10–20
Number of points irradiated	1
Irradiated area (cm^2^)	0.33
Distance from the laser tip (cm)	1
Number and frequency of treatment sessions	1 application (24 h)
Total energy delivered (J)	1–5–10–20

**Table 2:** Experimental design and treatment groups.

Group	Treatment Description
C	Negative control—cells not treated with ZA and not irradiated.
ZA	Cells treated with ZA at the ideal concentration determined in Section 2.2.
ZA+660nm-1J	Cells treated with ZA and irradiated at a wavelength of 660 nm with an energy dose of 1 J.
ZA+660nm-5J	Cells treated with ZA and irradiated at a wavelength of 660 nm with an energy dose of 5 J.
ZA+660nm-10J	Cells treated with ZA and irradiated at a wavelength of 660 nm with an energy dose of 10 J.
ZA+660nm-20J	Cells treated with ZA and irradiated at a wavelength of 660 nm with an energy dose of 20 J.
ZA+808nm-1J	Cells treated with ZA and irradiated at a wavelength of 808 nm with an energy dose of 1 J.
ZA+808nm-5J	Cells treated with ZA and irradiated at a wavelength of 808 nm with an energy dose of 5 J.
ZA+808nm-10J	Cells treated with ZA and irradiated at a wavelength of 808 nm with an energy dose of 10 J.
ZA+808nm-20J	Cells treated with ZA and irradiated at a wavelength of 808 nm with an energy dose of 20 J.

**Table 3:** Sequences of primers used to analyze gene expression of irradiated cells.

Gene	Oligonucleotide Sequence
BAX	Forward 5′-TGA AGA CAG GGG CCT TTT TG-3′ Reverse 5′ AAT TCG CCG GAG ACA CTC G-3′
BCL-2	Forward 5′-GGAGGCTGGGATGCCTTTGT Reverse 5′ AAAGCCAGCTTCCCCAATGA
GAPDH	Forward 5′-TCGACAGTCAGCCGCATCTTCTTT Reverse 5′ ACCAAATCCGT GACTCCGACCTT

## Data Availability

The data supporting the findings of this study are available from the corresponding author upon reasonable request.

## Abbreviations

The following abbreviations are used in this manuscript.

PBM	Photobiomodulation
ZA	Zoledronic acid
ONJ	Osteonecrosis of the jaw
BRONJ	Bisphosphonate-related osteonecrosis of the jaw
ATP	Adenosine triphosphate
ROS	Reactive oxygen species
NO	Nitric oxide
NF-κB	Nuclear factor κB
USA	United States of America
FBS	Fetal bovine serum
CO_2_	Carbon dioxide
C	Celcius
MTT	3-(4,5-dimethyltriazol-2yl)-2,5-diphenyl tetrazoline bromide
PBS	Phosphate-buffered saline
DMSO	Dimethylsulfoxide
OD	Optical density
t	Treatment
b	Blank
c	Control
RT-qPCR	Real-time polymerase chain reaction
RNA	Ribonucleic acid
DNA	Deoxyribonucleic acid
H_2_O	Water
